# The balance between cell cycle arrest and cell proliferation: control by the extracellular matrix and by contact inhibition

**DOI:** 10.1098/rsfs.2013.0075

**Published:** 2014-06-06

**Authors:** Claude Gérard, Albert Goldbeter

**Affiliations:** 1 Unité de Chronobiologie théorique, Faculté des Sciences, Université Libre de Bruxelles (ULB), Campus Plaine, CP 231, Brussels 1050, Belgium; 2 Stellenbosch Institute for Advanced Study (STIAS), Wallenberg Research Centre at Stellenbosch University, Marais Street, Stellenbosch 7600, South Africa

**Keywords:** cell cycle, focal adhesion kinase, extracellular matrix, contact inhibition, cyclin-dependent kinase oscillations, systems biology

## Abstract

To understand the dynamics of the cell cycle, we need to characterize the balance between cell cycle arrest and cell proliferation, which is often deregulated in cancers. We address this issue by means of a detailed computational model for the network of cyclin-dependent kinases (Cdks) driving the mammalian cell cycle. Previous analysis of the model focused on how this balance is controlled by growth factors (GFs) or the levels of activators (oncogenes) and inhibitors (tumour suppressors) of cell cycle progression. Supra-threshold changes in the level of any of these factors can trigger a switch in the dynamical behaviour of the Cdk network corresponding to a bifurcation between a stable steady state, associated with cell cycle arrest, and sustained oscillations of the various cyclin/Cdk complexes, corresponding to cell proliferation. Here, we focus on the regulation of cell proliferation by cellular environmental factors external to the Cdk network, such as the extracellular matrix (ECM), and contact inhibition, which increases with cell density. We extend the model for the Cdk network by including the phenomenological effect of both the ECM, which controls the activation of the focal adhesion kinase (FAK) that promotes cell cycle progression, and cell density, which inhibits cell proliferation via the Hippo/YAP pathway. The model shows that GFs and FAK activation are capable of triggering in a similar dynamical manner the transition to cell proliferation, while the Hippo/YAP pathway can arrest proliferation once cell density passes a critical threshold. The results account for the dependence or independence of cell proliferation on serum and/or cell anchorage to ECM. Whether the balance in the Cdk network is tilted towards cell cycle arrest or proliferation depends on the direction in which the threshold associated with the bifurcation is passed once the cell integrates the multiple, internal or external signals that promote or impede progression in the cell cycle.

## 1. Introduction

Understanding the dynamical bases of the cell cycle is crucial not only in normal physiological conditions, as it underlies development of the fertilized egg into an adult organism, but also in pathological ones, because deregulation of the cell cycle is associated with aberrant cell proliferation and cancer. In mammals, the cell cycle consists of four successive phases: in the S phase, the cell replicates its DNA, while in mitosis (M phase), the cell partitions its replicated DNA into two daughter cells. Two gap phases (G1 and G2) separate DNA replication from mitosis; G1 extends from mitosis to the next round of DNA replication, while G2 is the gap between S and the next M phase. A network of enzymes known as cyclin-dependent kinases (Cdks) governs the correct ordering of the cell cycle. Both S and M phases are controlled by reversible Cdk phosphorylation [1].

A Cdk is active as a protein kinase only when forming a complex with a regulatory subunit, called cyclin. In fission yeast, it was shown that a single cyclin/Cdk complex (cyclin B/Cdk1) can provide perfect temporal order in the eukaryotic cell cycle [2,3]. This led to the proposal of a ‘quantitative model’ of cell cycle control, according to which small Cdk activity is sufficient to trigger DNA replication, while higher activity is required to bring about M phase [2,3]. However, in higher eukaryotes, different cyclin/Cdk complexes are responsible for initiation of the S and M phases, which suggests a ‘qualitative model’ of cell cycle control [4]. This is especially true in mammals where a Cdk network consisting of at least four cyclin/Cdk complexes ensures the progression along the different phases of the cycle. Thus, cyclin D/Cdk4–6 and cyclin E/Cdk2 promote progression in G1 and elicit the G1/S transition; the activation of cyclin A/Cdk2 ensures progression in S and G2, while the peak of cyclin B/Cdk1 activity brings about progression into mitosis. Nonetheless, Cdk1 appears to be the major kinase, as it can bind to cyclins D, E and A, and replace Cdk4–6 and Cdk2 for correct progression in the cell cycle [5].

Mathematical models for the Cdk network based on experimental observations are helpful for investigating the dynamics of the cell cycle in view of the considerable complexity of this major cellular regulatory network. Owing largely to the pioneering work carried out over the last two decades by John Tyson and Béla Novák, together with their co-workers, the dynamics of the cell cycle has become a major, exemplary topic of research in computational cell biology. Computational models have thus been proposed for the regulatory network driving the cell cycle in embryos [6–8], yeast [9,10] and subsequently in mammals [11]. Several models were proposed to account for the dynamics of parts of the mammalian cell cycle, such as the G1/S transition [12–15], the restriction point in G1 that defines a point of no return beyond which cells do not need the presence of growth factor (GF) to complete a cycle [16], or the exit of mitosis [17]. Taking a global approach, we presented a detailed, 39-variable model [11,18] for the dynamics of the full mammalian cell cycle and later proposed a reduced, skeleton version derived from this model, which contains only five variables [19,20]. In these models, as recalled in §2, the transition from quiescence to proliferation corresponds to a critical bifurcation point at which a stable steady state, associated with cell cycle arrest, becomes unstable and sustained Cdk oscillations, corresponding to cell proliferation, develop.

Using the detailed model for the Cdk network driving the mammalian cell cycle, we previously investigated how the dynamics of the Cdk network is affected by GFs or factors internal to the network, such as the oncogenes E2F and Cdc25 as well as the tumour suppressors pRB, Cdh1 or Wee1 [11,18]. Here, using the same model, we focus on the control of cell proliferation by factors external to the Cdk network and address in turn the role of the extracellular matrix (ECM) and of cell contact inhibition. The ECM controls cell proliferation through the focal adhesion kinase (FAK) [21]. We first extend the model for the Cdk network by including in §3 the phenomenological effect exerted by ECM on the dynamics of the Cdk network via FAK. Cell proliferation is further controlled by cell density via contact inhibition, mediated by the Hippo/YAP pathway [22]. In §4, we incorporate this additional mode of control into the model for the Cdk network. Thus extended, the computational model for the mammalian cell cycle [11,18] illustrates how changes across threshold values in the stiffness of ECM or in cell density may tilt the balance towards either cell cycle arrest or cell proliferation. The model provides a unified framework to comprehend how this balance is tilted by factors external to the Cdk network, such as GFs, ECM stiffness or cell density, and by factors internal to the network, such as various oncogenes or tumour suppressors.

## 2. Tilting the balance between cell cycle arrest and cell proliferation in the cyclin-dependent kinase network

**Figure 1.**
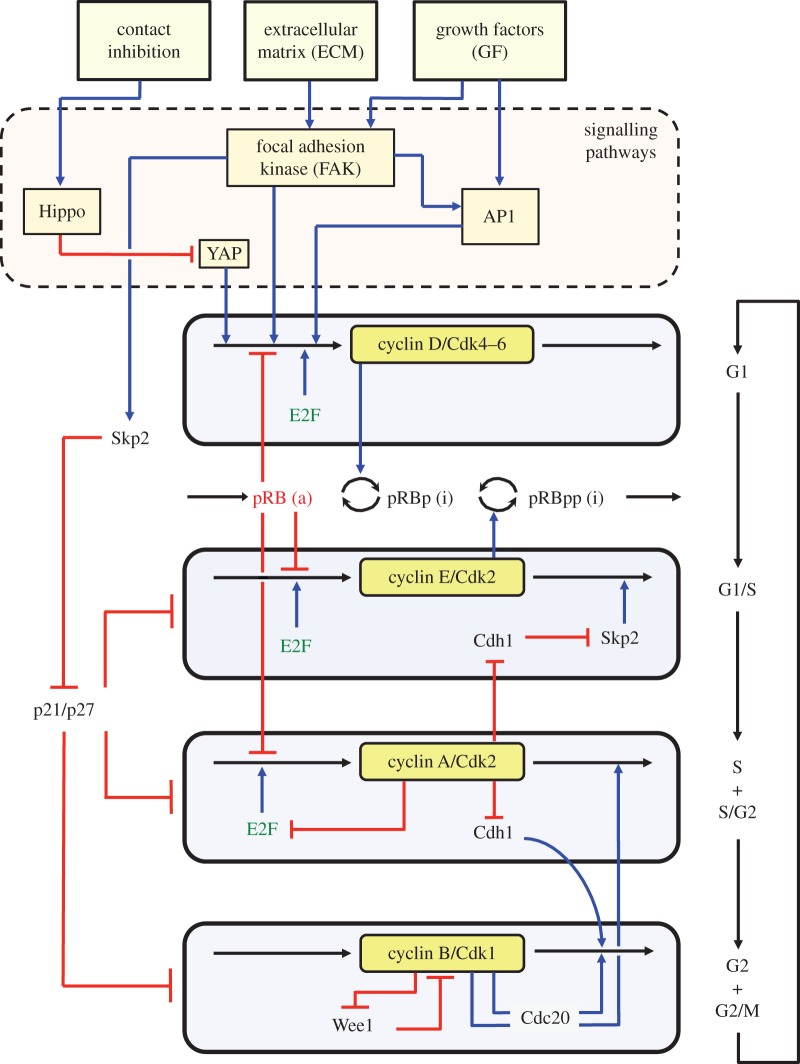
Extended model for the cyclin/Cdk network driving the mammalian cell cycle. The model presented in a simplified manner is composed of four modules centred on the main cyclin/Cdk complexes: cyclin D/Cdk4–6, cyclin E/Cdk2, cyclin A/Cdk2 and cyclin B/Cdk1, which control the successive phases G1, S, G2 and M of the cell cycle depicted on the right part of the figure. Entry from a quiescent phase G0 (not shown) into phase G1 of the cell cycle is driven by GFs and/or sufficient stiffness of the ECM. Defining a serum-dependent growth pathway, the presence of GFs elicits the activation of signalling pathways leading to the synthesis of AP1; this transcription factor in turn promotes the synthesis of cyclin D, which is followed by entry into G1. Defining an anchorage-dependent growth pathway, ECM stiffness activates the FAK, which eventually leads to the synthesis of cyclin D that elicits entry into the cell cycle. Entry in the cell cycle is impeded by contact inhibition at high cell density, via the Hippo/YAP pathway. Also indicated are the antagonistic roles of the transcription factor E2F and the tumour suppressor pRB, which, respectively, promote and impede cell cycle progression. Because it incorporates the external control of the Cdk network by the ECM via FAK, on one hand, and by cell density via Hippo and YAP, on the other hand, the model represents an extension of its previous version presented in [11]. Cyclin D/Cdk4–6 and cyclin E/Cdk2 drive progression in G1 and the G1/S transition by phosphorylating, and thereby inhibiting, pRB. Cyclin A/Cdk2 allows progression in S and G2, while cyclin B/Cdk1 brings about the G2/M transition. The active, unphosphorylated form of pRB inhibits E2F, which promotes cell cycle progression by inducing the synthesis of cyclins D, E and A. The protein Cdh1, inhibited by cyclin A/Cdk2, promotes the degradation of cyclin B, and inhibits Skp2, which promotes the degradation of cyclin E; activation of cyclin A/Cdk2 thus leads to the activation of cyclin B/Cdk1 and to the inhibition of cyclin E/Cdk2. The protein Cdc20, activated by cyclin B/Cdk1, promotes the degradation of cyclin A and cyclin B, which leads to the decrease in cyclin A/Cdk2 and cyclin B/Cdk1. The roles of the Cdk inhibitor p21/p27 and of the Cdk inhibitory kinase Wee1 are also indicated, together with the positive feedback loop involving Wee1 and Cdk1; the role of the phosphatase Cdc25 which activates Cdk1 and is activated by it, thus creating another positive feedback loop, is not indicated for the lack of space (see the electronic supplementary material in [11] for more detailed schemes of the model for the Cdk network). The regulatory interactions between the four Cdk modules give rise to sustained Cdk oscillations (figures 2–8), which allow the repetitive, ordered progression along the successive phases of the cell cycle (see also [11,18]). Electronic supplementary material, tables S1 and S2 provide a list of all variables and parameters considered in the model. (Online version in colour.)

In the detailed model for the mammalian cell cycle [11], which counts 39 variables and is schematized in figure 1, exit from the quiescent state is triggered, via the transcription factor AP1, by the synthesis of cyclin D, which allows cells to enter the G1 phase of the cell cycle. The synthesis of the various cyclins is regulated through the balance between the antagonistic effects exerted by the transcription factor E2F, which promotes, and the tumour suppressor pRB, which inhibits cell cycle progression. The Cdk network in turn regulates through phosphorylation the activities of E2F and pRB. Additional regulations in the detailed model for the Cdk network bear on the control exerted by the proteins Skp2, Cdh1 or Cdc20 on the degradation of cyclins E, A and B at the G1/S or G2/M transitions, respectively. Finally, the activity of each cyclin/Cdk complex can itself be regulated through Cdk phosphorylation–dephosphorylation. Thus, we consider that the activity of Cdk4–6 is activated by phosphorylation by the cyclin-activated kinase protein, while Cdk2 and Cdk1 are activated by the phosphatase Cdc25 and inhibited by the kinase Wee1. Multiple positive feedback loops control the activation of the Cdks, because the phosphatases Cdc25 that activate the various Cdks are themselves activated through phosphorylation by the Cdks, while the latter inactivate their inhibitory kinase Wee1 (see the Cdk1 module in figure 1 where only the positive feedback regulation of Wee1 is shown, while the positive feedback loop involving Cdc25 is not represented for the lack of space). The activity of the Cdks is further regulated through association with the protein inhibitor p21/p27, considered as a single entity in the model (see legend of figure 1 and [11] for further details).

The cyclin/Cdk network thus contains four modules centred on cyclin D/Cdk4–6, cyclin E/Cdk2, cyclin A/Cdk2 and cyclin B/Cdk1, respectively. We previously showed that the regulatory wiring of the Cdk network is such that each module is activated in turn in a transient manner, because a given module activates the subsequent modules and inhibits the previous ones. This temporal self-organization takes the form of sustained oscillations of the limit cycle type [11,18,19]. The periodic variations in the activity of cyclin/Cdk complexes are of a large amplitude owing to the fact that the positive feedback loops that control several of the Cdk modules give rise to bistability associated with all-or-none transitions between distinct levels of Cdk activity [20]. Such bistable switches contribute to make the transitions between successive phases of the cell cycle irreversible [23–25]. Stochastic simulations of models for the cell cycle suggest that the multiplicity of positive feedback loops not only provides redundancy in Cdk regulation, which could serve as a safeguard mechanism, but also contributes to enhance the robustness of Cdk oscillations with respect to molecular noise [20,26].

A variety of antagonistic factors affect the sensitive balance that controls the transition from cell cycle arrest to proliferation [27]. These factors include soluble GFs, as well as an abundance of oncogenes, which activate cell cycle progression, and tumour suppressors, which, on the opposite, inhibit cell proliferation and tumourigenesis. Additional external factors to be considered in §§ 3 and 4 are cell anchorage to extracellular surfaces, which depends on the stiffness of the ECM and contact inhibition, which increases with cell density (figure 1).

**Figure 2.**
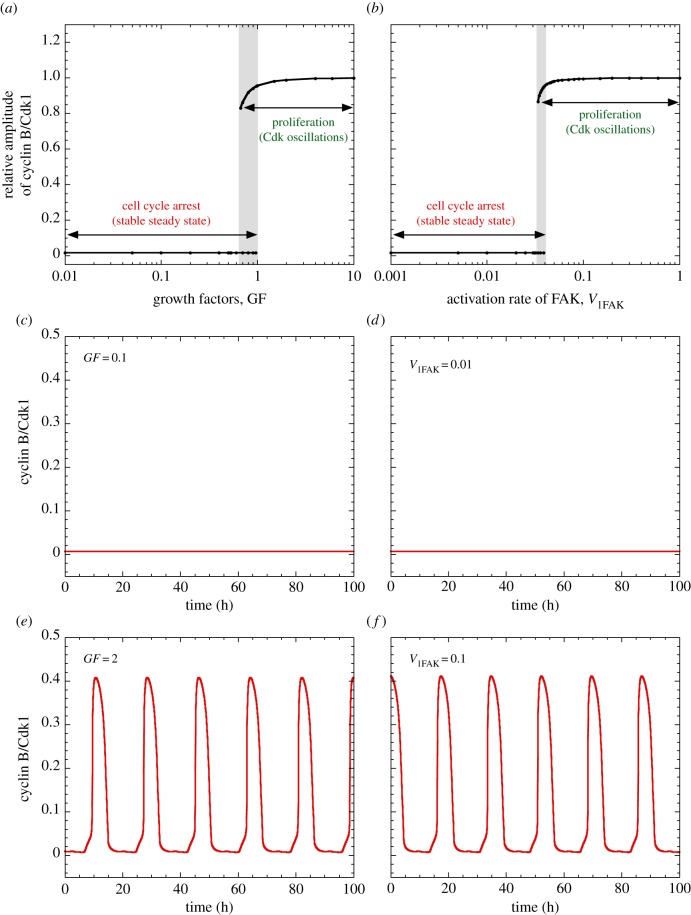
Switching between cell cycle arrest and proliferation upon increasing the level of GF or the activity of FAK. Shown in (*a*,*b*) are bifurcation diagrams established, respectively, as a function of GF and of FAK activation measured by parameter *V*_1FAK_. Low levels of GF as well as low activation rates of FAK elicit a low, stable steady-state level of cyclin/Cdk complexes represented by cyclin B/Cdk1. Such low levels correspond to cell cycle arrest, as shown by the time evolution of cyclin B/Cdk1 in (*c*), where *GF* = 0.1 μM and in (*d*), where *V*_1FAK_ = 0.01 h^−1^. By contrast, increasing above a threshold the level of GF or the activation rate of FAK triggers sustained, high-amplitude oscillations of the various cyclin/Cdk complexes, as illustrated for cyclin B/Cdk1 in (*e*), where *GF* = 2 and in (*f*), where *V*_1FAK_ = 0.1 h^−1^. In (*a*,*b*), the grey zone denotes a region of hard excitation in which a stable steady state coexists with sustained Cdk oscillations. In panels (*a*,*c*,*e*), *V*_1FAK_ = 0.04 h^−1^, while in panels (*b*,*d*,*f*), *GF* = 1 μM. As for subsequent figures, the curves are obtained by numerical integration of the kinetic equations (S1)–(S42) listed in the electronic supplementary material. To describe the activation exerted by FAK on the synthesis of cyclin D and Skp2 (see text), the term *k*_cd3_ · FAK has been added in the electronic supplementary material, equation (S12) describing the time evolution of cyclin D, while the term *V_s_*_2skp2_·FAK has been added to the electronic supplementary material, equation (S19) describing the time evolution of Skp2 (see the electronic supplementary material). In these simulations as well as in the simulations of figures 3–5, we only focus on the role of ECM and FAK and do not consider the effect of the Hippo/YAP pathway on cell proliferation. Thus, we fix the value *k*_cd4_ = 0 in the electronic supplementary material, equation (S12) for the time evolution of cyclin D, so that the latter is not induced by YAP (the effect of the Hippo/YAP pathway is illustrated in figures 6–8). Other parameter values are as in the electronic supplementary material, table S2. Initial conditions used in the different simulations are also indicated in the electronic supplementary material. (Online version in colour.)

The switch from cell cycle arrest to proliferation is illustrated by the bifurcation diagram established in figure 2*a* as a function of increasing levels of GFs [11]. At low levels of GF, this diagram shows the stable steady-state level of one of the 39 variables, cyclin B/Cdk1, representative of the whole Cdk network. This stable steady state corresponds to cell cycle arrest. Above a critical level of GF, the steady state becomes unstable and sustained oscillations develop in all variables of the network, including the various cyclin/Cdk complexes. Then, instead of the steady state, we plot in the bifurcation diagram of figure 2*a* the relative amplitude of cyclin B/Cdk1 in the course of oscillations, i.e. the level of cyclin B/Cdk1 at its peak, divided by the maximum peak height in the presence of high levels of GF. Such sustained oscillations correspond to the repetitive, sequential activation of the various Cdk complexes responsible for the ordered progression along the different phases of the cell cycle and can therefore be associated with cell proliferation. The stable steady state of cyclin B/Cdk1 below the critical level of GF is shown in figure 2*c*, while its oscillatory time course above the GF threshold is displayed in figure 2*e*. In a narrow range of GF values (grey zone in figure 2*a*), the stable steady state coexists with stable oscillations. Such a phenomenon, known as hard excitation, is observed in a number of models for periodic behaviour in biochemical and cellular systems [28].

The detailed model for the Cdk network driving the mammalian cell cycle can be used, much as for the effect of GF, to analyse the response of the network towards changes in the levels of activators and inhibitors of cell cycle progression. Examples of activators, which often behave as oncogenes, are cyclins [29–31], the transcription factor E2F [32] and the phosphatases Cdc25 that activate the Cdks by dephosphorylation [33]. Inhibitors, which behave as tumour suppressors, include pRB [34], the Cdk inhibitor p21/p27 [35], the protein Cdh1 involved in cyclin degradation [36] and the kinase Wee1 responsible for Cdk inhibition through phosphorylation [37]. In each case, decreasing below a threshold the level of activators/oncogenes leads to cell cycle arrest, characterized by a low, stable steady-state activity of the different cyclin/Cdk complexes. Conversely, starting from such a stable steady state, the increase in the level of any of the various activators/oncogenes above a critical value triggers the onset of Cdk oscillations and, hence, cell proliferation. The switch between cell cycle arrest and proliferation is governed by the balance of activators versus inhibitors. We previously showed that Cdk oscillations can repetitively be induced, and then suppressed, by sequential, alternating increments in E2F and pRB [11]. Therefore, the switch from steady state to oscillations in the dynamics of the Cdk network appears to be governed by the relative rather than absolute levels of activators/oncogenes and inhibitors/tumour suppressors.

The model predicts, in a somewhat counterintuitive manner, that a sufficiently large increase in the level of activators, such as Cdk1, the cyclins or the phosphatases Cdc25, might lead to cell cycle arrest, characterized by a stable steady state corresponding to high Cdk activity, because oscillations often occur in a range bounded by two critical values of the control parameter (not shown). The question arises as to how to interpret the nature of this hyperactivated-Cdk steady state. Evolution to such a stable steady state of the Cdk network, corresponding to cell cycle arrest, would not necessarily represent cellular quiescence. The evolution to such a state could also lead to senescence (the cell is blocked at the G1/S transition and GFs cannot induce re-entry into the cell cycle [38]), or to programmed cell death, i.e. apoptosis [39]. That sustained high levels of Cdk1 are capable of inducing apoptosis has been reported experimentally [40]. For this reason, we prefer to refer to the stable steady state of the Cdk network as a state of cell cycle arrest rather than a quiescent state, as quiescence and return to the G0 phase is but one of the possible outcomes of cell cycle arrest.

## 3. Control of cell proliferation by the extracellular matrix and focal adhesion kinase

Besides GFs and the factors internal to the network considered in the preceding section, an additional way to tilt the balance controlling the dynamics of the Cdk network involves the ECM, which mediates the interactions of the cell with its physical environment. Healthy cells need the presence of a proper microenvironment and sufficient stiffness in the ECM to proliferate [41]. Sensing of stiffness and anchorage of the cell to ECM are integrated by a complex network of integrin signalling pathways [42], which involves numerous molecular components among which the FAK is a key actor [42,43]. Activation of FAK through several signalling pathways leads to the activation of ERK and MEK kinases, which eventually promote cyclin D synthesis, thereby allowing entry into the G1 phase of the cell cycle; see simplified scheme in figure 1, and [44,45]. Besides its control of cell proliferation, FAK is also an important regulator of cell motility [46]. Here, we extend the model proposed for the dynamics of the Cdk network driving the mammalian cell cycle [11] by considering the effect of the ECM on entry into the cycle.

Because our goal is to model the action of FAK on the dynamics of the Cdk network, we consider, for simplicity, that FAK is directly activated by the stiffness of ECM. Soluble GFs can also activate FAK through the GF receptor signalling pathways [42,43]. Pathways controlled by FAK include the cascade of activation involving Ras, Raf, MEK and ERK, and lead to the expression of immediate-early genes, such as Jun/Fos, also called AP1. Thus, AP1 can be turned on by both FAK and GFs (figure 1). The transcription factor AP1 induces the synthesis of cyclin D [47]. Here, we retain a phenomenological description of the effect of ECM and consider, for simplicity, that FAK directly elicits the synthesis of both cyclin D [44,48–51] and AP1 [42], disregarding the details of the signalling pathways downstream of FAK.

To incorporate such regulations into the model, we add a term for synthesis dependent on FAK in the kinetic equation describing the time evolution of AP1 (see the electronic supplementary material, equation (S1), where a list of all equations considered in the extended model is presented). Incorporated is a new kinetic equation (electronic supplementary material, equation (S2)), which describes the time evolution of the active form of FAK. Parameters in the electronic supplementary material, equations (S1) and (S2), are defined, along with other parameters of the model, in the electronic supplementary material, table S2. The stiffness of ECM is represented in the electronic supplementary material, equation (S2), by a parameter, *ECM*, which controls the activation of FAK through phosphorylation–dephosphorylation: the enzyme is first activated via autophosphorylation; this results in the activation of the kinase Src, which further phosphorylates and activates FAK [52]. We assume for simplicity that FAK phosphorylation is simply proportional to *ECM* and consider that this parameter measures at the same time the stiffness of ECM and the anchorage capability of the cell, given that both increase in concert. Finally, in agreement with experimental observations [53], we consider that FAK promotes the synthesis of Skp2, which leads to the degradation of p27 (figure 1). These regulations are incorporated into the kinetic equation governing the time evolution of Skp2 (see the electronic supplementary material, equation (S19)).

The extended model for the Cdk network includes two modes of entry into the cell cycle: as considered in the original version [11], GFs promote the synthesis of AP1, leading to the synthesis of cyclin D; in addition, the stiffness and anchorage of the cell to the ECM allow phosphorylation and activation of FAK, which also promotes the synthesis of cyclin D (figure 1). Increasing the rate of FAK activation (measured by parameter *V*_1FAK_ in the electronic supplementary material, equation (S2)) above a threshold is by itself capable of inducing the transition from cell cycle arrest to proliferation (figure 2*b*) in much the same way as a supra-threshold increase in the level of GF (figure 2*a*). The bifurcation diagram is highly similar (compare figure 2*b* with figure 2*a*): as for GF, a stable steady state associated with cell cycle arrest is reached below a critical threshold of FAK activation (figure 2*d*), while sustained Cdk oscillations associated with cell proliferation occur above the FAK activation threshold (figure 2*f*).

**Figure 3.**
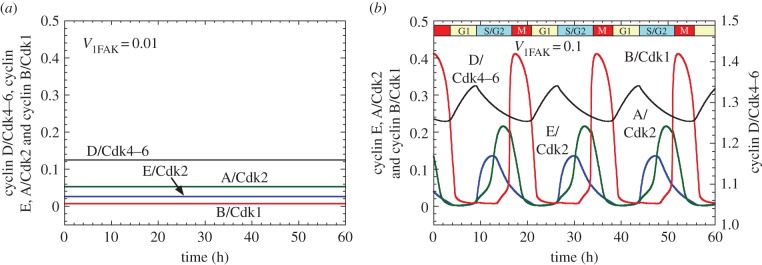
Effect of FAK activation on the onset of oscillations in the Cdk network driving the mammalian cell cycle. The time course of the active forms of cyclin D/Cdk4–6, cyclin E/Cdk2, cyclin A/Cdk2 and cyclin B/Cdk1 is shown in the presence of (*a*) low (*V*_1FAK_ = 0.01 h^−1^) and (*b*) high activation of FAK (*V*_1FAK_ = 0.1 h^−1^). Low FAK activation leads to a stable steady state in the levels of the various cyclin/Cdk complexes, which corresponds to cell cycle arrest. High FAK activation elicits the sequential, periodic activation of the four Cdk modules; this oscillatory behaviour of the Cdk network can be associated with cell proliferation. Conditions in (*a*,*b*) are the same as in figure 2*d*,*f*, respectively. (Online version in colour.)

The oscillations, displayed only for cyclin B/Cdk1 in figure 2*f*, are illustrated in figure 3*b* for the four cyclin/Cdk complexes, whereas the evolution of these complexes to a stable steady state is shown in figure 3*a* at subthreshold levels of FAK activation in the same conditions as in figure 2*d*. If attachment of healthy cells to the ECM is needed for correct cell proliferation, it is also crucial for cell survival. Indeed, the loss of cell–ECM adhesion can lead to cell death through anoikis [54]. Therefore, the stable steady state observed in figure 3*a* at subthreshold levels of FAK activation, which corresponds to low cell adhesion to ECM, likely defines a state of cell death.

**Figure 4.**
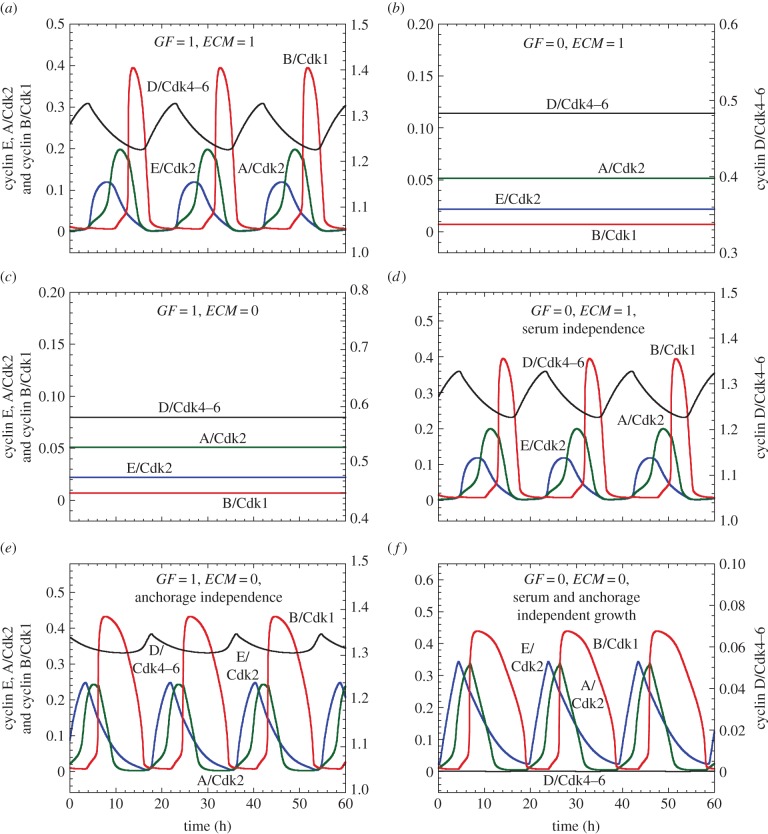
Regulation of the Cdk network by ECM and GF: serum- and anchorage-dependent or independent growth. The time evolution of cyclin D/Cdk4–6, cyclin E/Cdk2, cyclin A/Cdk2 and cyclin B/Cdk1 is shown in the presence (*GF* = 1 μM in (*a*,*c*,*e*)) or absence of soluble GFs (*GF* = 0 in (*b*,*d*,*f*)), and in the presence (*ECM* = 1 in (*a*,*b*,*d*)) or absence of ECM stiffness (*ECM* = 0 in (*c*,*e*,*f*)). (*a*) Healthy cell proliferation, characterized by the repetitive, sequential activation of the various cyclin/Cdk complexes, depending on GF and on the stiffness in ECM. From that condition, removing either GF (*GF* = 0 in (*b*)) or the stiffness in ECM (*ECM* = 0 in (*c*)) elicits cell cycle arrest. (*d*) Increasing the rate of activation of FAK, *V*_1FAK_, from 0.04 to 0.1 h^−1^ leads to cell proliferation even without GF, resulting in serum-independent cell growth. Conversely, an increase in the rate of synthesis of AP1, *V*_sap1_, from 0.05 to 1 μM h^−1^ allows cell proliferation without stiffness in ECM, leading to anchorage-independent growth (*e*). Overexpression of the transcription factor E2F by increasing its rate of synthesis, *V*_se2f_, from 0.15 to 0.5 μM h^−1^ elicits cell proliferation without GF nor stiffness in ECM (*GF* = 0 and *ECM* = 0), which situation defines serum- and anchorage-independent growth. Other parameter values are as in the electronic supplementary material, table S2. (Online version in colour.)

Healthy cells need both the presence of GFs in the serum and proper anchorage of the cell to the ECM to enter into a proliferative state. This situation defines an AND gate between GF and ECM for entry into the cell cycle; in contrast, transformed and cancer cells can exhibit serum- and/or anchorage-independent growth [55]. The extended model for the Cdk network allows us to consider both cases. Let us first deal with the case of healthy cells (figure 4*a*–*c*). In the presence of GF and cell anchorage to ECM (e.g. *GF* = *ECM* = 1), cells enter into a proliferative mode characterized by sustained oscillations of the various cyclin/Cdk complexes (figure 4*a*). The cells evolve to a stable steady state either when the GF is absent (figure 4*b*, *GF* = 0, *ECM* = 1) or when cell anchorage is missing or the stiffness of the ECM is too low (figure 4*c*, *GF* = 1, *ECM* = 0). By contrast, when the balance is strongly tilted towards cell proliferation—e.g. by hyperactivation of FAK in figure 4*d*, overexpression of AP1 in figure 4*e* or overexpression of E2F in figure 4*f*—cells proliferate in the absence of GF (figure 4*d*, *GF* = 0, *ECM* = 1), or when cell anchorage is missing or the stiffness of the ECM is too low (figure 4*e*, *GF* = 1, *ECM* = 0), or when both GFs and cell anchorage are missing (figure 4*f*, *GF* = 0, *ECM* = 0).

The model therefore suggests that overactivation of FAK in the absence of GF (figure 4*d*) or overexpression of AP1 in the absence of anchorage to ECM (figure 4*e*) can lead to serum- or anchorage-independent growth, respectively. This result supports experimental observations showing the role of FAK and AP1 in serum- and anchorage-independent cell growth [56]. In particular, a recent study indicates that overexpression of AP1 upregulates cyclin D, leading to anchorage-independent cell growth [57]. The model further shows that the overexpression of oncogenes, such as the transcription factor E2F, can elicit anchorage- and serum-independent growth (figure 4*f*), which is a characteristic of cancer cells [32,58].

**Figure 5.**
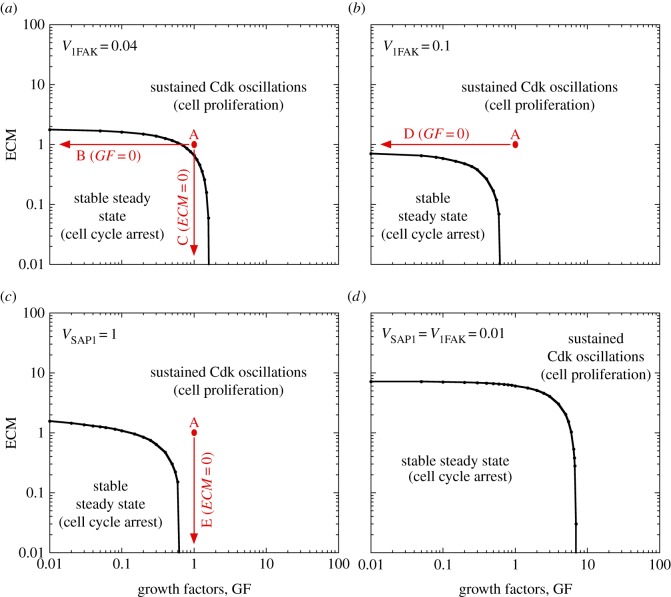
Balance between cell cycle arrest and cell proliferation as regulated by ECM and GF. Dynamical behaviour of the Cdk network driving progression in the mammalian cell cycle is shown in the parameter plane defined by *ECM* and *GF*. (*a*) *V*_1FAK_ = 0.04, which corresponds to the situation in figure 4*a*–*c*. (*b*) *V*_1FAK_ = 0.1, which corresponds to the conditions of figure 4*d* with *GF* = 0. (*c*) *V*_SAP1_ = 1, which matches the conditions of figure 4*e* with *ECM* = 0. (*d*) Low levels of *V*_1FAK_ and *V*_SAP1_ are considered: *V*_1FAK_ = *V*_SAP1_ = 0.01. This reduction in the rates of activation of AP1 and FAK considerably extends the domain of cell cycle arrest. Dots and arrows in red correspond to the different situations illustrated in figure 4*a*–*e*. Other parameter values are as in figure 4. (Online version in colour.)

The dependence of the switch between cell cycle arrest and cell proliferation on the level of GF and ECM stiffness can be recapitulated by representing the dynamical behaviour of the Cdk network in the parameter plane defined by ECM and GF for different rates of activation of FAK (*V*_1FAK_) or different rates of synthesis of AP1 (*V*_SAP1_) (figure 5). Cell proliferation only occurs at sufficiently large levels of either ECM or GF. By increasing *V*_1FAK_, the domain of cell proliferation is extended (compare figure 5*a* with figure 5*b*). A low level of *V*_1FAK_ and *V*_SAP1_ considerably enlarges the domain of cell-cycle arrest (figure 5*d*). The transitions between the different situations considered in figure 4*a*–*e* are indicated by vertical or horizontal arrows in the two-parameter state diagrams in figure 5.

## 4. Control of cell proliferation by contact inhibition and the Hippo/YAP pathway

Besides FAK, which serves to integrate proliferation signals transduced from ECM into the cell, other pathways play a role in modulating cell proliferation as a function of the extracellular environment. An important physiological process in this regard is contact inhibition of cell proliferation at high cell density, which is mediated by cadherin adhesion molecules via the Hippo signalling pathway. This pathway plays a crucial role in cell contact inhibition, organ size control and cancer development [59–62]. It inhibits cell growth through a kinase cascade that leads to the phosphorylation and nuclear exclusion of the growth-promoting transcriptional co-activator YAP (figure 1).

To study the effect of cell density on cell proliferation, we incorporate the role of the Hippo pathway into the model for the Cdk network. Retaining a phenomenological approach similar to that followed above for the control of cell proliferation by ECM and FAK, we reduce the complexity of the contact inhibition signalling pathway by focusing on Hippo and YAP. We assume that Hippo is activated by intercellular contacts, which are mediated by cadherin molecules present at the cell surface and which increase with cell density, while YAP is phosphorylated downstream of Hippo and thereby kept in the cytosol in a form unable to elicit synthesis of cyclin D [59–62]. Based on these regulatory properties, we describe the time evolution of Hippo and YAP by two new kinetic equations listed in the electronic supplementary material, equations (S3) and (S4). In the model, we consider that contact inhibition, measured by parameter *CI*, elicits the activation of Hippo (see the electronic supplementary material, equation (S3) for the evolution of Hippo). In turn, Hippo can inhibit YAP (see the electronic supplementary material, equation (S4) for the evolution of YAP), while the active form of YAP can activate the synthesis of cyclin D. This is expressed by adding a term of synthesis proportional to YAP in the rate equation of cyclin D (see the electronic supplementary material, equation (S12)).

**Figure 6.**
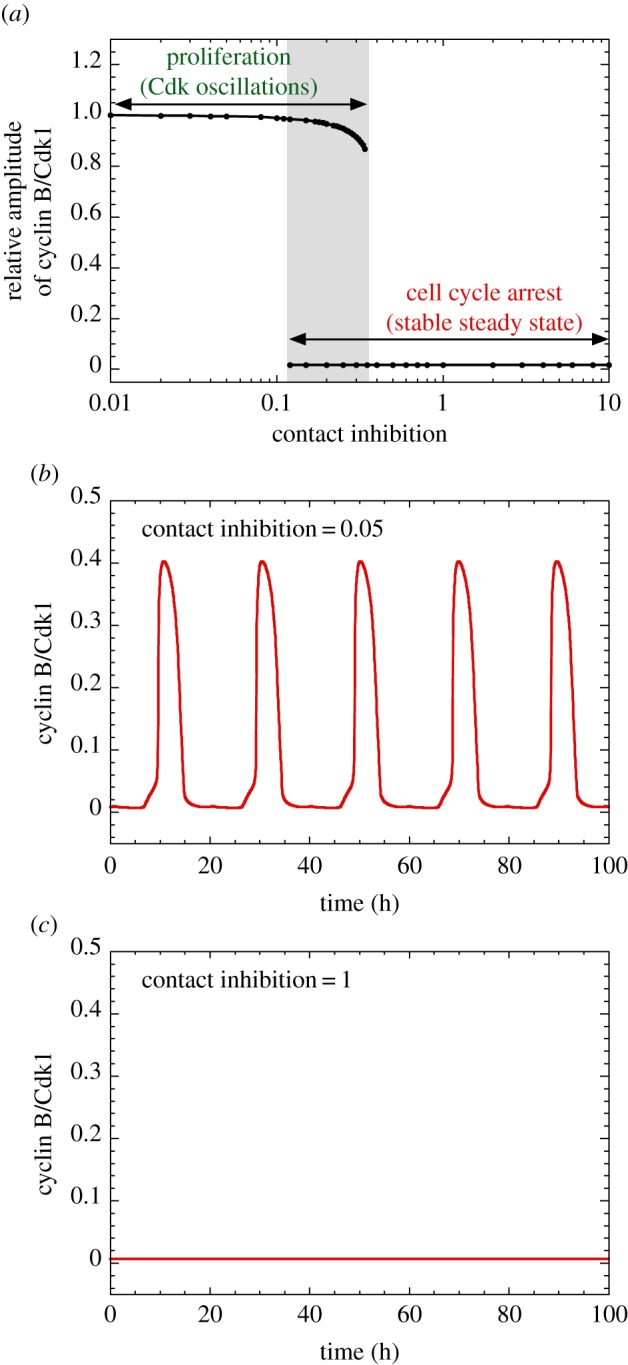
Switching from proliferation to cell cycle arrest by increasing contact inhibition via the Hippo/YAP pathway. (*a*) Bifurcation diagram showing the relative level of cyclin B/Cdk1 as a function of contact inhibition. A low level of *CI*, corresponding to low cell density, ensures cell proliferation, while a supra-threshold level, corresponding to high cell density, completely inhibits cell proliferation. Time series of cyclin B/Cdk1 is illustrated for a low value of *CI* in (*b*) (*CI* = 0.05), which ensures sustained oscillations of cyclin/Cdk complexes, and in (*c*) for higher contact inhibition (*CI* = 1), which impedes cell proliferation. Parameter values are as in the electronic supplementary material, table S2 with *k*_cd3_ = *k*_cd4_ = 1 h^−1^. The relative level of cyclin B/Cdk1 is the ratio of the maximum level of Cdk1 in the different conditions to its maximum level when *CI* = 0. (Online version in colour.)

Much as for the activation of cell proliferation by ECM via FAK, but in the opposite direction, an increase in cell density above a critical threshold can tilt the balance from cell proliferation towards a stable steady state corresponding to cell cycle arrest. This negative control is illustrated in figure 6*a* by the bifurcation diagram showing the level of cyclin B/Cdk1 as a function of the strength of contact inhibition, which increases with cell density. Sustained Cdk oscillations corresponding to proliferation occur below the contact inhibition threshold (figure 6*b*), while above the threshold the dynamical behaviour of the Cdk network reaches a stable steady state corresponding to cell cycle arrest (figure 6*c*). As in the bifurcation diagrams of figure 2*a*,*b* established as a function of GF and FAK, respectively, we observe in figure 6*a* a range of hard excitation in which Cdk oscillations coexist with a stable steady state.

**Figure 7.**
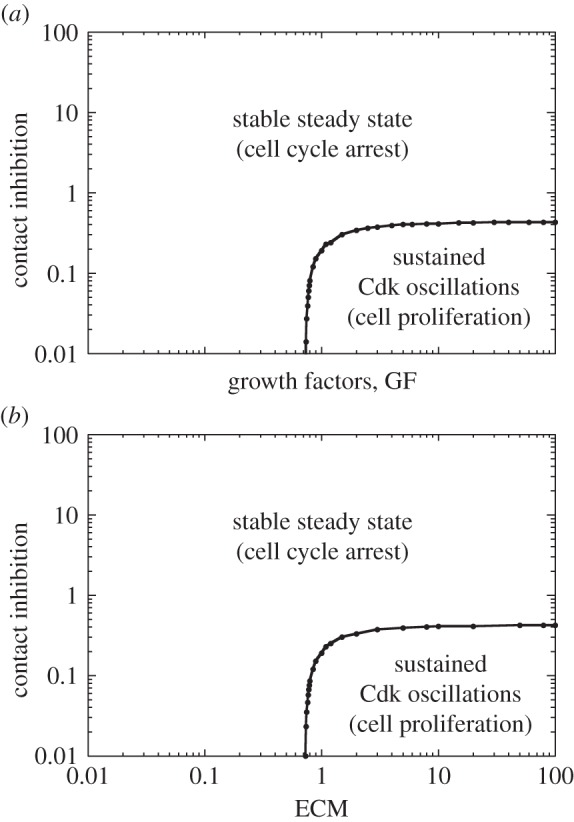
Balance between cell cycle arrest and cell proliferation regulated by ECM, GF and contact inhibition. The dynamical behaviour of the Cdk network is determined in the parameter plane defined by *CI* and *GF* in (*a*), and by *CI* and *ECM* in (*b*). Parameter values are as in figure 6.

**Figure 8.**
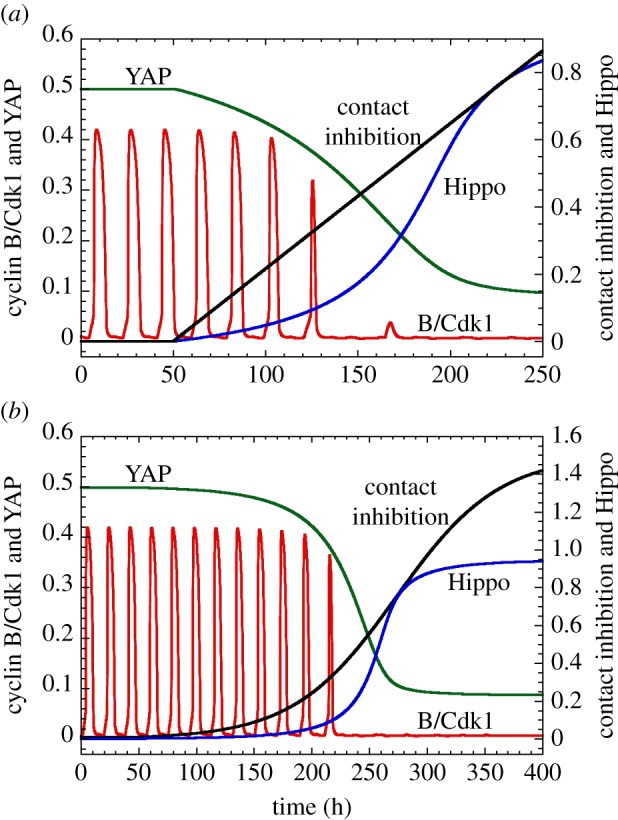
Inhibition of cell proliferation by increasing cell density, via the Hippo/YAP pathway. Shown is the time evolution of cyclin B/Cdk1 and of the active forms of Hippo and YAP, when the level of contact inhibition increases (*a*) linearly or (*b*) according to a logistic equation. In both cases, cell proliferation is first slowed down and then arrested at sufficiently high contact inhibition. Between the first and last cycles, the period of the cell cycle passes from 18.5 to 22.3 h in (*a*) and from 18.5 to 21.6 h in (*b*). In (*a*), the linear increase in *CI* starts from *t* = 50 h, with a slope of 0.0043. In (*b*), the time evolution of *CI* is governed by the logistic law d*CI*/d*t* = 0.015 *CI* (1.5−*CI*). Contact inhibition leads to cell cycle arrest when *CI* > 0.45, which corresponds to Hippo larger than 0.25 and YAP smaller than 0.2. Parameter values are as in figure 7 with *k*_cd1_ = 0.1 h^−1^, *k*_cd3_ = 0.2 h^−1^ and *k*_cd4_ = 3 h^−1^. (Online version in colour.)

The dynamical behaviour of the Cdk network in response to external signals can conveniently be represented in the parameter planes defined by the strength of contact inhibition and by either *GF* (figure 7*a*) or *ECM* (figure 7*b*). These diagrams indicate that cell proliferation occurs for sufficiently large levels of *GF* or *ECM* as long as *CI* remains smaller than a critical value. The model also shows that, from a condition of active cell proliferation, a progressive increase in the strength of contact inhibition, by a linear (figure 8*a*) or logistic law (figure 8*b*), slows down cell proliferation before leading to cell cycle arrest.

## 5. Discussion

A sensitive balance between a variety of inhibitors and activators of progression in the cell cycle regulates the switch between cell cycle arrest and cell proliferation [27]. Inhibitory factors include numerous tumour suppressors, anti-GFs, differentiation factors and signals triggered by low stiffness of the ECM or high cell density. Activating factors comprise a variety of oncogenes and GFs as well as proper cellular microenvironment characterized by sufficient stiffness of ECM allowing anchorage of the cell [27,48].

The detailed computational model for the Cdk network driving the mammalian cell cycle, that we previously proposed [11] and extend here, allows us to gain further insight into the finely tuned balance between factors that promote and impede progression in the cell cycle. The model suggests that the switch from cell cycle arrest to proliferation is associated with the transition from a stable steady state to sustained oscillations in the activity of the various cyclin/Cdk complexes that control the successive phases of the cell cycle.

We showed earlier that the transition can be induced through changes in the levels of GFs or in the levels of activators (such as oncogenes) or inhibitors (such as tumour suppressors) present within the Cdk network. Here, we expanded our previous analysis [11,18] by focusing on the control of cell proliferation by external cues, such as the stiffness of the ECM or cell density. We first examined the roles of the ECM and FAK that transduce the information about the stiffness of the microenvironment to the biochemical machinery controlling cell proliferation. Then, we turned to the control of cell proliferation by contact inhibition, which builds up with cell density, and which involves the Hippo/YAP signalling pathway. The results allow us to integrate and unify various ways to tilt the balance between cell cycle arrest and cell proliferation by factors internal or external to the Cdk network.

Mammalian cells are embedded in a cellular microenvironment, which is composed of a complex protein network, the ECM, which can exert important control over cell proliferation. Such control is mediated by integrins, cell surface proteins that transduce mechanical and chemical signals from the matrix into the cell and allow its binding to the support. In turn, these signals regulate the cytoskeleton as well as a complex cascade of intracellular kinases, which can promote different types of cellular responses, such as cell proliferation or its arrest. Cell cycle arrest may in fact be followed by one of several possible outcomes: quiescence, senescence, apoptosis, motility or differentiation [42]. The FAK is a key signalling component, capable of integrating a variety of stimuli to control cell proliferation and motility [43]. A large number of cells, such as fibroblasts and epithelial cells, must be properly anchored to ECM to survive and proliferate. This anchorage requirement is very often lost in transformed and cancer cells [56,63,64]. The latter pathological condition may be correlated in part with a deregulation of FAK [43,65].

To study the effect of ECM on the dynamics of the Cdk network, we extended our detailed model for the mammalian cell cycle by including the role of FAK in the regulation of entry into the cell cycle (figure 1). The results show that the supra-threshold activation of FAK can trigger the switch from cell-cycle arrest to proliferation much in the same way as for a supra-threshold increase in the level of GFs. In both cases, the supra-threshold increase in FAK activity (figure 2*b*) or in GF (figure 2*a*) elicits the transition from a stable steady state, associated with cell cycle arrest, to sustained oscillations in the Cdk network, associated with cell proliferation. That the dynamical effects of FAK and GF on the cell cycle are similar stems from the fact that both elicit AP1 synthesis in equivalent ways (see the electronic supplementary material, equation (S1)), and therefore promote in a similar manner cyclin D synthesis and the subsequent entry into the cell cycle.

The extension of the model for the Cdk network allowed us to consider the anchorage- and serum-dependence of cell proliferation. The model accounts for the observation that healthy cells need the presence of both anchorage to ECM and serum to proliferate (figure 4*a*–*c*). The model further shows how cell proliferation might become serum- or anchorage-independent, as a result, for example, of overactivation of FAK (figure 4*d*) or overexpression of AP1, a key downstream output of GF receptor signalling pathways that promotes cyclin D synthesis (see figure 4*e* as well as [57]). Moreover, an overexpression of oncogenes that act within the Cdk network, such as E2F, could elicit anchorage- and serum-independent growth, as observed in cancer cells (figure 4*f* and [32,58]).

Integrating into the model the Hippo/YAP pathway, which mediates the limiting effect of cell density on cell proliferation, allowed us to determine an additional mode of control of the Cdk network by an external signal. In contrast to the effect of ECM stiffness, which is to favour cell proliferation, cell density inhibits it once it exceeds a critical value. While the various effectors of the balance between cell cycle arrest and proliferation have been treated separately so far, it is important to note that there may exist an interplay between different signalling pathways linking cell adhesion and proliferation. An example is provided by the interplay of cell contact inhibition, mediated by the Hippo pathway, with mitogenic factors, such as EGF, which inhibits Hippo signalling [62]. Hippo and YAP also appear to control in a cell-density-dependent manner the expression of microRNAs and, thereby, the synthesis of the transcription factor MYC, which stimulates cell proliferation [66].

As cells are not disconnected from their environment and are embedded in a complex ECM, perturbations of signals mediated by ECM, for example as a result of chronic inflammation [67,68] or by contact inhibition [60–62] could drive the transition from a healthy to a cancer cell. Such a transition frequently originates from perturbations associated with mutations that change inside the cell the activity of tumour suppressors or oncogenes [27,29–37]. Although the two mechanisms based, respectively, on tissue organization and genetic mutations are sometimes viewed as contradictory [69], the present computational approach provides a unifying framework that indicates how the two types of perturbations, which both influence the dynamics of the Cdk network, can bring about in a similar manner its abusive activation leading to uncontrolled cell proliferation.

A recent experimental study showed that the decision between quiescence and proliferation is controlled by Cdk2 activity at mitotic exit [70]. Such dynamical behaviour has been proposed to result from a bistable switch that originates from a positive feedback loop in the Cdk2-RB-E2F module [71]. The model for the Cdk network considered here indicates that in a certain range of mitotic signals, cell proliferation may coexist with a state of cell cycle arrest (figure 2*a*). In that range, the dynamics of the Cdk network is very sensitive to the initial level of Cdk2. Small random fluctuations in the level of Cdk2 may then favour the switch from quiescence to cell proliferation (results not shown). This result could bear on the observation that Cdk2 activity can act as bifurcation parameter in the transition from quiescence to proliferation [70].

**Figure 9.**
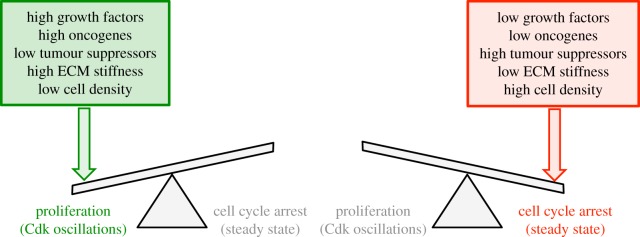
The balance towards cell cycle arrest or proliferation can be tilted by multiple antagonistic effectors. An abrupt switch in the dynamics of the Cdk network that controls progression in the mammalian cell cycle occurs in a bifurcation point separating a stable steady state, associated with cell cycle arrest (right), from a regime of spontaneous, sustained Cdk oscillations associated with cell proliferation (left) [11,18]. To decide towards which side the balance will be tilted, the cell integrates the multiple signals that promote (GFs, oncogenes, high ECM stiffness and low cell density) or impede (tumour suppressors, low ECM stiffness and high cell density) cell proliferation. (Online version in colour.)

We showed that the switch from cell cycle arrest to proliferation can be achieved by tilting beyond a critical threshold value a finely tuned balance between the antagonistic effects of multifarious factors that, more or less directly, impinge on the Cdk network that drives the mammalian cell cycle. The computational approach to the dynamics of this complex network provides a unifying, integrated view of how the balance between cell cycle arrest and proliferation can be tilted through changes in the levels of GFs, oncogenes and tumour suppressors, or through modifications in cell density or in the stiffness of the ECM (figure 9). In its current state, the model does not incorporate some of the most important oncogenes, such as Myc, Ras, Raf, and some of the most prevalent tumour suppressors, such as p53, PTEN Arf and Ink4. Such additional oncogenes or tumour suppressors could be included in the model and are expected to exert similar antagonistic effects on the global balance that governs the dynamics of the Cdk network.

The model for the cyclin/Cdk network is based on regulatory interactions at the level of proteins. The present extension brings the number of variables up to 42 (not counting five additional variables needed to describe the DNA-replication checkpoint [11], which was not retained here for the sake of simplicity). The complexity of the model would be further increased by incorporating the role of a large number of microRNAs as well as the regulation of mRNA synthesis and degradation, which were also shown to control the dynamics of the cell cycle [72–74].

By extending the model for the Cdk network to the control exerted by the ECM and contact inhibition, and linking the results to those previously obtained for the effect of GFs, oncogenes and tumour suppressors, we showed that all these factors, internal or external to the Cdk network, converge through multiple signalling pathways to induce the crossing of the bifurcation point towards either cell cycle arrest or proliferation, depending on their combined effect. The bifurcation from a stable steady state to sustained oscillations in the Cdk network appears to be governed by the relative rather than absolute levels of these antagonistic effectors. Towards which side the balance is tilted depends on the direction in which the bifurcation point associated with the threshold is crossed once the cell integrates the multiple signals that promote or impede cell proliferation.
